# Comparison of hydrocortisone and prednisone in the glucocorticoid replacement therapy post-adrenalectomy of Cushing's Syndrome

**DOI:** 10.18632/oncotarget.20597

**Published:** 2017-08-31

**Authors:** Kunlong Tang, Liang Wang, Zhongyuan Yang, Yingying Sui, Liming Li, Yuting Huang, Peng Gao

**Affiliations:** ^1^ Tianjin Medical University General Hospital, Tianjin, China; ^2^ Sun Yat-sen University Cancer Center, Guangzhou, China; ^3^ Tianjin Medical University Cancer Hospital and Institute, Tianjin, China; ^4^ University of the District of Columbia, Washington, DC, USA

**Keywords:** Cushing’s syndrome, glucocorticoid replacement, adrenocortical adenoma, Cushing's disease

## Abstract

Cushing's syndrome requires glucocorticoid replacement following adrenalectomy. Based on a simplified glucocorticoid therapy scheme and the peri-operative observation, we investigated its efficacy and safety up to 6 months post-adrenalectomy in this cohort study.

We found the adrenocorticotropic hormone (ACTH) levels were normal post-adrenalectomy, and sufficient to stimulate the recovery of the dystrophic adrenal cortex, thus exogenous supplemental ACTH might not be necessary.

Patients were grouped by oral reception of either hydrocortisone or prednisone since day 2 post-adrenalectomy. Both groups had similar baseline responses to adrenalectomy, regarding the correction of hypertension (10/15 vs.12/19), hyperglycemia (6/11 vs. 7/10), and hypokalemia (12/12 vs. 11/11). Most patients lost weight (17/20 vs. 20/22). Both groups reported significant improvement in a subjective evaluation questionnaire. Hydrocortisone showed advantages over prednisone in improving liver function (7/8 vs. 2/7, *p* = 0.035), but also caused significant lower extremety edema (*p* = 0.034).

Both groups developed adrenal insufficiency (AI) during glucocorticoid withdrawal, with no significant difference regarding the incidence rate (7/20 vs. 10/22) or severity. Most AI symptoms were relieved by resuming the prior oral doses, while two severe cases were hospitalized. The withdrawal process may last longer time for hydrocortisone than prednisone.

In conclusion, our data supports the use of both hydrocortisone and prednisone in the glucocorticoid replacement therapy post-adrenalectomy for patients of adrenal adenoma or Cushing's disease. Hydrocortisone showed advantages over prednisone in improving liver function, and prednisone exhibited significantly lower risk of edema.

## INTRODUCTION

The symptoms of Cushing's syndrome can be largely related to mineralocorticoid produced by zona glomerulosa, glucocorticoid secreted by zona fasciculata, and sexual corticoid produced by zona seticularis. A sharp drop of these hormone levels after adrenalectomy may result in acute adrenal crisis. Therefore post-arenalectomical glucocorticoid replacement therapy is required for patients of Cushing's syndrome [1, 2]. Cushing's syndrome, also named hypercortisolism, is a heterogeneous condition with distinct causes, including Cushing's disease (CD) or pituitary tumors in most cases, and less often ectopic ACTH syndrome or adrenocortical cancer. However there lacks evidence based guideline for the use of glucocorticoid, and individual institutes may have developed their own schema [3]. The current Chinese guideline differs from the guideline of Endocrine Society. The recommended glucocorticoid is explicitly hydrocortisone in the Endocrine Society guideline [3] and prednisone in the Chinese guideline [4]. Although hydrocortisone has been widely used for a long time, it is yet less prescribed in China, probably due to problems in its domestic availability and high cost of the oral tablet. Such prescription preference is also seen in the hormone replacement therapy of other diseases. For example, among patients with congenital adrenal hyperplasia (CAH), prednisolone/prednisone and hydrocortisone are prescribed to the almost the same amount of patients [5, 6]. In UK, 20% female and 39% male CAH patients are on hydrocortisone, while prednisolone covers 49% female and 29% male patients respectively [7]. As both hydrocortisone and prednisone have demonstrated their efficacy in decades of practice, few studies have compared them directly to address whether one has significant advantages over the other [8]. Therefore we included both Cushing's syndrome patients on hydrocortisone and prednisone in our study for comparison.

Also, the recommended therapy in the Chinese guideline involves several drawbacks [9]: (1) glucocorticoid is delivered intravenously followed by subcutaneously, which practice may increase the risk of ecchymosis as the patients’ blood vessels are often fragile; (2) the schema of glucocorticoid administration are too complicated. To address the above issues, we have explored a simplified scheme for oral glucocorticoid replacement therapy, by removing the pre-operative glucocorticoid administration and changing the post-adrenalectomical subcutaneous and intravenous delivery to oral tablet [9, 10].

We have previously published the peri-operative results [10] of this simplified scheme with oral hydrocortisone or prednisone in respect to the major measures of efficacy. A 6-month follow-up of the patients is reported regarding its safety and secondary therapeutic benefits.

## MATERIALS AND METHODS

### Enrollment criteria and acquisition of clinical information

This study included cohort of Cushing's syndrome patients admitted to Tianjin Medical University General Hospital between 2010 October and 2013 June. Inclusion criteria were: (1) ACTH independent Cushing's syndrome patients who had undergone adrenalectomy, or (2) CD patients who had undergone adrenalectomy at least 3 months after hypophysectomy. The following exclusion criteria were applied: (1) treated without adrenalectomy; (2) with other diseases that require hormone replacement therapy; (3) with neurological or psychiatric disease history; (4) with alcohol addiction; (5) with hepatic pre-conditions such as hepatitis. Patients were randomly grouped for the use of hydrocortisone or prednisone.

The cohort started with 45 patients, of which 2 were lost in follow-up, and 1 died of adrenocortical cancer 3 months after adrenalectomy. The rest 42 patients were included in the statistical analysis (Table 1). The collected information included age, sex, body weight, location and size of lesion, length of disease, blood chemistry and hormone panel, liver panel, medical imaging results, *etc*.

**Table 1:** Description of the included patients

	Prednisone		Hydrocortisone	
	adenoma	CD	adenoma	CD
**Pathology**				
Adrenocortical adenoma	15/20	1/20	13/22	1/22
Adrenoadenoma-like nodules	1/20	1/20	1/22	2/22
Adrenal nodular hyperplasia	0/20	2/20	0/22	5/22
**Endocrinology**				
LDDST no inhibition	10/10	4/4	14/14	8/8
HDDST no inhibition	10/10	1 /4	14/14	3/8
ACTH elevation	0/16	4/4	0/14	8/8
**Average age**	37.1 (20–59)	45.3 (28–54)	41.5 (20–54)	39.6 (29–51)
**Lengths of disease (months)**	24.6 (1–96)	32.8 (5–96)	18.2 (0.5–72)	22.4 (11–36)

CD, Cushing's disease; LDDST, low dose dexamethasone suppression test; HDDST, high dose dexamethasone suppression test; ACTH, adrenocorticotropic hormone.

When admitted, blood was collected in EDTA at 0 am, 8 am, 4 pm and analyzed by radioimmunoassays to determine the concentration of cortisol and its diurnal rhythm. For the first 3 days of admission, 24 h urine was collected and combined every day to measure the urinary cortisol concentration by radioimmunoassay, and blood was collected in EDTA to measure ACTH concentration.

Patients were given 0.5 mg dexamethasone orally every 6 hours for 2 days. An inhibition was defined if dexamethasone led to at least 50% reduction of the cortisol concentration in the 24 h urine of the first 2 days. In total 36 patients underwent low dose dexamethasone suppression test (LDDST). For high dose dexamethasone suppression test (HDDST), the dose escalated to 2 mg every 6 hours.

The hypertension patients were on their medications before operation, and hypokalemia patients were supplemented with potassium orally (40 mg t.i.d. in tablet or in 10% solution) or by intravenous infusion. Hyperglycemia was controlled by oral hypoglycemic medication or subcutaneous injection of insulin.

All patients underwent retroperitoneal laparoscopic adrenalectomy [11]. The 30 adrenal adenoma patients were performed with 19 unilateral partial adrenalectomy and 11 unilateral total adrenalectomy. The CD patients included 3 unilateral total adrenalectomy after hypophysectomy, 2 contralateral subtotal adrenalectomy after hypophysectomy and an initial unilateral subtotal adrenalectomy, and 7 patients without identifiable pituitary pathology, including 2 unilateral total adrenalectomy and contralateral subtotal adrenalectomy, and 5 unilateral adrenalectomy.

All patients received no glucocorticoid before adrenalectomy. The intravenous doses of hydrocortisone were 100 mg during adrenalectomy, 100 mg t.i.d. on day 0 and day 1, followed by 100 mg q.d. on the morning of day 2 post-adrenalectomy. Oral glucocorticoid replacement started on day 2, at a dose of 10 mg t.i.d. prednisone with decrement of 5 mg/week, or 40 mg t.i.d. hydrocortisone with decrement of 10 mg/week. The withdrawal schedule varied by individual patients.

Patients were followed up at 1 month, 3 months, and 6 months post-adrenalectomy. They were monitored for vital signs and indices related to their symptoms, including fasting plasma glucose (FPG), blood electrolytes, blood cortisol, and 24 h urinary cortisol. Liver functions were primarily monitored by ALT and AST, both with thresholds of 40 U/L. During the telephone interview, the actual glucocorticoid withdrawal process and incidence of adrenal insufficiency (AI) were followed up, and the interviewer also conducted questionnaire (Supplementary Table 1) [12].

### Statistical methods

Numeric values of this study were presented in mean ± SEM unless specified, using Graphpad Prism. The variables of time series were compared by student *t*-test (unpaired, two-tailed), and the numbers of patients with complete recovery of hepatic panel, FPG, hypertension, and the incidence of edema, were compared by chi-square test with threshold *p* < 0.05 for statistical significance.

## RESULTS

### Sufficient endogenous ACTH secretion

**Figure 1:**
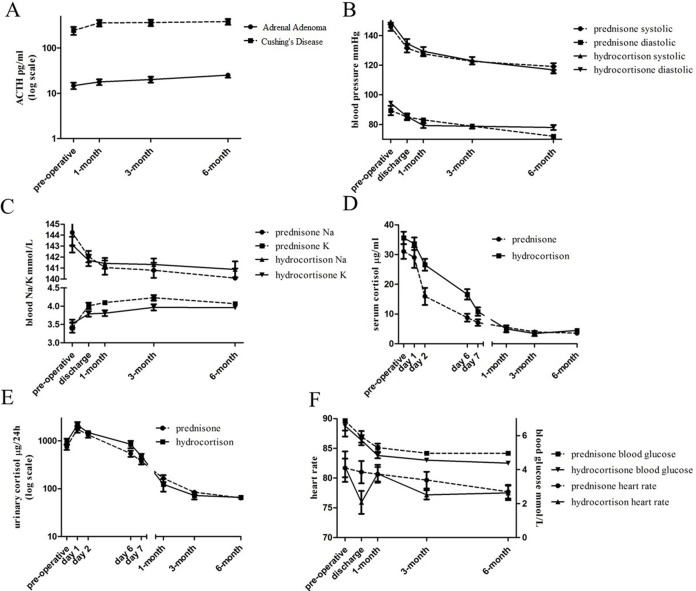
Similar baseline responses to adrenalectomy between patients given prednisone and hydrocortisone (**A**) ACTH levels increased after adrenalectomy in patients of adrenal adenoma and Cushing's disease. (**B**, **C**) Blood and urinary cortisol levels decreased steadily post adrenalectomy. (**D**) Increased blood potassium and decreased blood sodium levels. (**E**) Improved blood pressure profiles. (**F**) reduced heart rate (left) and reduced blood glucose (right) levels.

At 1 month, 3 months, and 6 months post-adrenalectomy, both the ACTH dependent CD patients and ACTH independent adrenocortical adenoma patients showed significant increase over time in endogenous ACTH levels (Figure 1A, Supplementary Table 2), in the absence of exogenous ACTH administration. Although the ACTH level of CD patients was still pathologically high (380.6 ± 136.0 pg/ml by 6 months), it started to recover in adrenal adenoma patients after adrenalectomy (25.2 ± 11.3 pg/ml by 6 months).

### Similar baseline responses to adrenalectomy

To make a fair comparison between the two glucocorticoids, we first analyzed the patients’ responses to adrenalectomy to exclude any confounding effects. In general, both groups of patients had similar responses to adrenalectomy, and most symptoms were relieved to different extent. Both blood and urinary cortisol levels dropped continuously to the normal levels by 6 months post-adrenalectomy (Figure 1B, 1C, Supplementary Table 3).

Abnormal mineralocorticoid production can cause anomalies of blood sodium, potassium, as well as hypertension. There were 12/20 and 11/22 patients in the two groups with hypenatremia or hypokalemia when admitted. Blood sodium dropped at all examined time points after adrenalectomy, and all patients had normal blood sodium levels after 1 month (Figure 1D, Supplementary Table 5). Blood potassium levels increased gradually, and were in the normal range by discharge for all patients (Figure 1D). However fluctuations of blood sodium and potassium were seen during the individual withdrawal process.

When admitted, 15/20 and 19/22 patients in the two groups had hypertension. These patients showed reduced systolic pressure and diastolic pressure at day 1, discharge, 1 month, 3 months, and 6 months post-adrenalectomy (Figure 1E, Supplementary Table 4). By 6 months, 10 patients of prednisone group and 12 patients of hydrocortisone group resumed normal blood pressures and stopped hypertension medication (Table 4). The rest of the hypertension patients also exhibited improved blood pressure profile, and continued medication with reduced doses. Patients on prednisone showed slightly better improvement of blood pressure than patients on hydrocortisone. Prednisone is milder than hydrocortisone in water and sodium preservation and potassium excretion, thus may facilitate the reduction in blood volume and blood pressure. But the actual recovery rates of the two groups of hypertension patients did not differ significantly.

Despite fluctuations at the above time points, we did not observe significant difference in heart rate (Figure 1F, Supplementary Table 6) before and after adrenalectomy and during glucocorticoid replacement therapy.

Common complications that relates to glucocorticoid include hyperglycemia and overweight. Prior to the adrenalectomy, 11/20 patients of prednisone group and 10/22 patients of hydrocortisone group were with FPG ≥ 6.1 mmol/L. FPG generally decreased over time after adrenalectomy (Figure 1F, Supplementary Table 6). Among the 11 hyperglycemic patients on prednisone 6 stopped medication while 7/10 hyperglycemic patients on hydrocortisone also stopped (Table 4). The rest of the patients who continued the medication also benefited from the alleviated FPG.

There was no significant change in bodyweight or body mass index (BMI) of the patients, though 14 of them slightly lost weight by discharge. However by 6 months, the weight loss became significant (Table 2). Among patients on prednisone, the mean bodyweight dropped to 62.6 kg in comparison to 68.3 kg at admission, and mean BMI dropped to 23.1 kg/m^2^ from 25.1 kg/m^2^. Similar trends were seen in patients on hydrocortisone, with bodyweight reduced from 68.6 kg to 61.0 kg and BMI reduced from 27.4 kg/m^2^ to 24.2 kg/m^2^.

**Table 2:** Change in bodyweight over time

	Prednisone		Hydrocortisone	
	Bodyweight kg	BMI kg/m^2^	Bodyweight kg	BMI kg/m^2^
admission	68.25 ± 10.74	25.13 ± 2.76	68.83 ± 9.60	27.40 ± 3.46
6 months	62.60 ± 5.60^*^	23.17 ± 1.79^*^	60.96 ± 5.99^*^	24.19 ± 2.16^*^

BMI, body mass index.

^*^indicates statistical significance (*p* < 0.05), paired student-*t* test.

**Figure 2:**
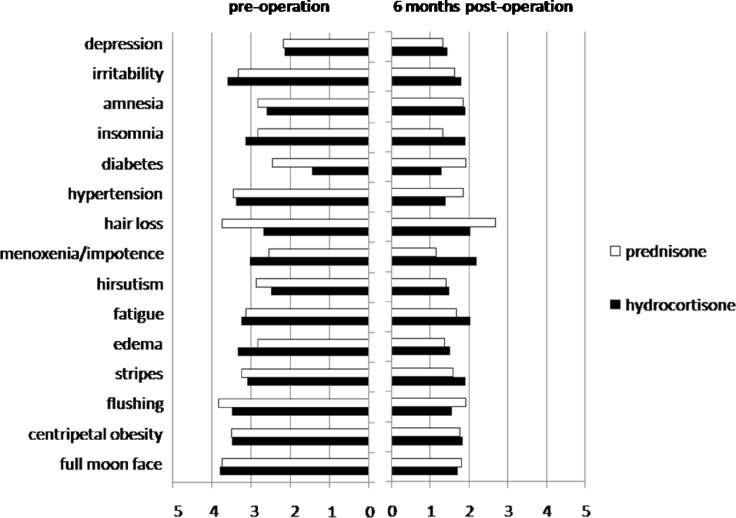
Grading on the relief of symptoms Common symptoms of Cushing's syndrome were graded by individual patients on a 0–5 scale before and 6 months after adrenalectomy. White bars indicate the average scores for patients on prednisone post-adrenalectomy, and black bars are for patients on hydrocortisone.

We adopted a questionnaire from previous study [12] to evaluate the patients’ subjective responses to adrenalectomy, rating primarily for indices of clinical symptoms, complications, and psychiatric conditions. The grading scale was between 1 and 5, whereas the score increased with the severity. By 6 months, all patients reported relief of symptoms (Figure 2), with 14/20 and 16/22 patients rated “significant”, 4/20 and 5/22 reported “decent”, 2/20 and 1/22 graded “slight” improvement for prednisone and hydrocortisone respectively (Table 3).

**Table 3:** Subjective evaluation of the overall efficacy

Rating of improvements	Prednisone	Hydrocortisone
Significant improvement (score change ≧ 20)	14 (70%)	16 (73%)
Decent improvement (10≦ score change < 20)	4 (20%)	5 (23%)
Slight improvement (1≦ score change < 10)	2 (10%)	1 (4%)
Exacerbation	0 (0%)	0 (0%)

### Incidence of adrenal insufficiency

No acute adrenal crisis was observed during the peri-operative period for prednisone and hydrocortisone groups, indicating the efficacy of glucocorticoid replacement therapy.

It is noteworthy during the withdrawal phase of the glucocorticoid replacement therapy, 7/20 and 10/22 patients of the two groups had AI condition with onset between day 11 and day 120 post-adrenalectomy (Table 5, Table 6). One case in each group was resulted from nonadherence to medication. Common symptoms of AI include nausea, fatigue, anorexia, pruritus, and lethargy *etc*. In each group, there was one patient with severe symptom that required immediate hospitalization for intravenous glucocorticoid infusion. For most AI patients, their symptoms disappeared after resuming the latest dose.

**Table 4:** Number of patients with improved liver function/FPG/blood pressure, or incidence of edema

	Prednisone	Hydrocortisone	*p*
Liver function	2/7	7/8	0.035^*^
FPG	6/11	7/10	0.659
Blood pressure	10/15	12/19	0.561
Lower extremity edema	0/20	6/22	0.034^*^

FPG, fasting plasma glucose.

^*^indicates statistical significance (*p* < 0.05), chi-square test.

**Table 5:** Incidence of adrenal insufficiency in prednisone group

Pathology	Age	Sex	Post-adrenalectomy	stage of withdrawal	Blood cortisol	Urinary cortisol	Treatment
**Adrenal Adenoma**							
	54	F	15 d	Nonadherence	1.1	54.4	5 mg b.i.d.
	29	F	35 d	10 mg q.d.→5 mg q.d.	1.2	49.5	Hospitalized
	49	F	45 d	5 mg b.i.d.→5 mg q.d.	< 1	30	5 mg b.i.d.
	45	F	75 d	5 mg t.i.d.→5 mg b.i.d.	< 1	NA	5 mg t.i.d.
	28	F	75 d	5 mg t.i.d.→5 mg b.i.d.	< 1	25	5 mg t.i.d.
**Cushing's disease**							
	37	F	75 d	5 mg t.i.d.→5 mg b.i.d.	< 1	22.4	5 mg t.i.d.
	50	F	120 d	1 week post cessation	NA	NA	5 mg q.d.

**Table 6:** Incidence of adrenal insufficiency in hydrocortisone group

pathology	Age	Sex	Post-adrenalectomy	stage of withdrawal	Blood cortisol	Urinary cortisol	treatment
**Adrenal Adenoma**							
	20	F	11 d	Nonadherence	< 1	< 21	Hospitalized
	47	F	25 d	40 mg b.i.d.→40 mg day/20 mg night	2.4	53	40 mg b.i.d.
	49	F	32 d	20 mg b.i.d.→20 mg q.d.	1.2	39.1	20 mg b.i.d.
	60	F	45 d	20 mg b.i.d.→20 mg q.d.	< 1	28	20 mg b.i.d.
	30	F	50 d	20 mg day/10 mg night→20 mg q.d.	1.4	56.2	20 mg day/10 mg night
	32	F	100 d	20 mg q.d.→10 mg q.d.	2.0	22	20 mg q.d.
	51	F	150 d	10 mg b.i.d.→10 mg q.d.	3.3	19	10 mg b.i.d.
**Cushing's disease**							
	54	F	52 d	20 mg b.i.d.→20 mg day/10 mg night	NA	NA	20 mg b.i.d.
	29	F	56 d	20 mg day/10 mg night→10 mg b.i.d.	< 1	< 11	20 mg b.i.d.
	34	M	75 d	20 mg b.i.d.→10 mg b.i.d.	< 1	NA	20 mg b.i.d.

### Advantage for hydrocortisone in liver function and less edema for prednisone

In the 20 patients given prednisone, 7 had abnormal liver function (ALT and/or AST over 40 U/L) and only 2 of them became normal by end of the study. In contrast, 7 out of 8 patients with abnormal liver function completely recovered after glucocorticoid therapy with hydrocortisone (Table 4). The rest of the patients also experienced relief of their hepatic symptoms to different extent if not complete recovery.

Meanwhile, 6/22 patients in the hydrocortisone group developed lower extremity edema at different stages of therapy. Two peri-operative edema cases developed on day 6 and day 7 respectively, with blood sodium 142–148 mmol/L, and blood potassium 3.6–3.9 mmol/L. The edema remitted after diuretics treatment. During the withdrawal process, another 4/22 patients exhibited lower extremity edema. Their blood sodium was 142–148 mmol/L, and blood potassium 3.4–3.8 mmol/L. Potassium sparing diuretic treatment with spironolactone 40 mg t.i.d. relieved all symptoms. Hypokalemic condition was seen in 6 patients in the hydrocortisone group during withdrawal, and the average blood potassium was 3.05 mmol/L (2.7–3.4 mmol/L). They were given oral potassium chloride. No edema or hypokalemia case was seen in the prednisone group during peri-operative period or the withdrawal stage.

## DISCUSSION

Despite the completely different pathology of CD and adrenocortical adenoma, both diseases exhibit the overproduction of corticoid and subsequent symptoms. Therefore they share some common therapeutic goals, including [13]: (1) resection of any harmful tumors; (2) reducing the corticoid secretion to normal level; (3) avoidance of permanent endocrine deficiency and glucocorticoid replacement therapy. For this reason, our study included both patients of CD and adrenocortical adenoma.

Our simplified glucocorticoid delivery scheme differs from the conventional schema in several aspects. We first eliminated the preoperative dose of glucocorticoid, as the necessity of such practice was unclear when considering the endogenous corticoid levels had been very high before adrenalectomy. Its efficacy and safety need to be monitored in long term. Another problem faced by the conventional glucocorticoid replacement therapy is the subcutaneous and intravenous delivery routes. Patients often suffer from problems like thinner than average skins and fragile vasculature. Subcutaneous injection or intravenous infusion may exacerbate the risk of ecchymosis. Our simplified scheme avoided subcutaneous injection and maximally replace the intravenous injection by oral delivery.

High level of corticoid steroid can cause long term suppression of the hypothalamic-pituitary-adrenal (HPA) axis and the subsequent dystrophy of the originally non-disease cortex. There has been a controversy over whether ACTH administration is necessary after surgical removal of the disease cortex. To provide more evidence to resolve this issue, we examined the levels of endogenous ACTH before and after adrenalectomy. ACTH administration was a common post-surgical practice in 1990s to rescue the dystrophic cortex once under HPA axis suppression. However, as ACTH is often produced from animal sources and requires subcutaneous or intravenous delivery, it may increase the risk of infection. Our data indicated the patients’ endogenous ACTH level increased after adrenalectomy. As the secretion of the patients’ endogenous ACTH production was sufficiently strong to rescue the dystrophic non-disease cortex, exogenous ACTH administration was unnecessary.

We monitored both blood and urine for cortisol concentration, and found urinary cortisol could better reflect the function of adrenal cortex. At 3 months and 6 months, some patients showed blood cortisol concentration lower than reference and normal urinary cortisol concentration, but none of them developed AI. Only free cortisol can be filtered by glomerular and enter the urine. Therefore urinary cortisol is not affected by cortisol binding globulin or the intrinsic fluctuation of blood cortisol [14]. This may explain the discrepancy between its concentrations in blood and urine.

Hydrocortisone and prednisone may fit patients with different complications. Hydrocortisone has short term retention of 8–12 hours, high bioavailability, low impact on blood glucose, and does not require hepatic metabolism [15], thus may be of priority for patients with diabetic or hepatic complications. In contrast, the action of prednisone may reach its peak at 50 minutes after administration and persist for 12–36 hours, with greater impact on blood glucose and hepatic metabolism [15]. Considering the possible hepatic impact by prednisone, future study may include prednisolone that does not require hepatic metabolism [16]. Glucocorticoid with long term action such as dexamethasone and betamethasone are not recommended for long term use due to their strong suppression on HPA axis.

The differences between hydrocortisone and prednisone may result in several important observations in our study, including impacts on blood glucose, liver function, electrolytes balance, incidence of edema, and duration of the glucocorticoid replacement therapy.

By 1 month and 3 months, hydrocortisone group had slightly higher blood/urinary cortisol than prednisone group (*p* > 0.05), and this situation reversed by 6 months. We consider the early lower cortisol concentration reflected the fast action and the short half-life of hydrocortisone, while the later but higher cortisol concentration implied better recovery of HPA axis and the autonomous glucocorticoid production in the hydrocortisone group.

Both groups did not differ in the incidence rate of AI. AI occurred in 7 out of 20, and 10 out of 22 patients during the reduction phase of glucocorticoid replacement therapy, though not seen in the first week after the procedure. Nevertheless patients on hydrocortisone spent longer time in glucocorticoid withdrawal. For adenoma patients, their endogenous ACTH had been low for long before the adrenalectomy, and the non-disease cortex often became dystrophic. Such condition implies adrenocortical adenoma patient should be particularly careful for AI during glucocorticoid withdrawal, as the recovery of the dystrophic cortex takes time. At the same time, patients on hydrocortisone may also carefully watch for AI. Hydrocortisone is metabolized faster than prednisone, thus symptoms may develop soon if exogenous glucocorticoid dose is insufficient.

Due to the limited number of enrolled patients, this study failed to cover the complete spectrum of Cushing's syndrome. Ectopic ACTH secretion due to lung cancer or pancreatic cancer can also cause Cushing's syndrome in some cases. After the surgical removal of the primary lesions, whether adrenalectomy is required and how to apply glucocorticoid replacement therapy are yet to be explored. It is also noteworthy one adrenocortical cancer patient was lost during the study by 3 months post-adrenalectomy. How adrenolectomy and the subsequent glucocorticoid replacement therapy affected the survival and the recurrence is also of clinical interest. Whether our conclusion could be extended to the case of adrenocortical cancer patients remains further study in specific cohorts.

In conclusion, our data supports the use of both hydrocortisone and prednisone in the glucocorticoid replacement therapy post-adrenalectomy in patients of adrenocortical adenoma or Cushing's disease. Hydrocortisone showed advantages over prednisone in improving liver function, and prednisone exhibited significantly lower risk of edema. Based on our observations, we call for a revision of the Chinese guidelines to include hydrocortisone. The use of hydrocortisone has been widely adopted and now written in the Endocrine Society guidelines. On the other hand, prednisone can be considered as an alternative to hydrocortisone, particularly for patients who develop edema during the withdrawal process and patients with hypernatremia or hypokalemia when admitted.

## SUPPLEMENTARY TABLES


