# Supplementary material for: Comparison of hydrocortisone and prednisone in the glucocorticoid replacement therapy post-adrenalectomy of Cushing's Syndrome
Source: Oncotarget. 2017 Aug 31;8(62):106113–20. doi: 10.18632/oncotarget.20597 (PMC5739705; doi:10.18632/oncotarget.20597)
Supplement: Supplementary file 1 [file oncotarget-08-106113-s001.pdf]

Comparison of hydrocortisone and prednisone in the glucocorticoid replacement therapy post-adrenalectomy of Cushing's Syndrome

SUPPLEMENTARY MATERIALS

Supplementary Table 1: Interview questionnaire

Name		Before adrenalectomy					After adrenalectomy				
Tel		1	2	3	4	5	1	2	3	4	5
Symptoms	Full moon face										
	Centripetal obesity										
	Flushing										
	Stripes										
	Edema										
	Fatigue										
	Hirsutism										
	Menoxenia/ Impotence										
	Hair loss										
	Subtotal										
Complications	Hypertension										
	Diabetes										
	Subtotal										
Psychiatry	Insomnia										
	Amnesia										
	Irritability										
	Depression										
	subtotal										
Total											
Quality of Life		Very satisfied		Rather satisfied		satisfied		unsatisfied		Highly unsatisfied	
Overall satisfaction		Very satisfied		Rather satisfied		satisfied		unsatisfied		Highly unsatisfied	
Height		Cm									
Bodyweight		Kg					Kg				

Grading: 1. No condition; 2. Insiginificant condition; 3. With condition; 4. Significant condition; 5. Very significant condition.

Supplementary Table 2: Serum ATCH concentration (pg/ml) post-adrenalectomy

	Adrenal Adenoma (*n* = 14)	CD (*n* = 6)
admission	14.8 ± 9.1	243.6 ± 121.6
1 month	17.8 ± 9.7▲	356.8 ± 143.1▲
3 months	20.2 ± 11.1▲	363.4 ± 143.4▲
6 months	25.2 ± 11.3▲	380.6 ± 136.0▲

Supplementary Table 3: Cortisol levels post-adrenalectomy

Stage	Prednisone		Hydrocortisone	
	Blood cortisol ug/ml	Urine cortisol ug/24 h	Blood cortisol ug/ml	Urine cortisol ug/24 h
admission	31.06 ± 11.21	747.16 ± 446.1523	35.57 ± 9.96	962.15 ± 649.42
day 1	29．00 ± 15.39	1752.99 ± 1243.81▲	33.65 ± 9.82	2136.40 ± 1444.02▲
day 2	15.94 ± 12.93▲	1331.58 ± 783.07▲	26.56 ± 9.29▲	1488.81 ± 572.21▲
day 6	8.78 ± 5.94▲	541.60 ± 363.22	16.61 ± 8.23▲	847.97 ± 673.63
day 7	7.12 ± 4.78▲	372.50 ± 230.76▲	10.82 ± 6.54▲	476.27 ± 264.02▲
1 month	5.54 ± 1.45▲	164.48 ± 120.48▲	5.01 ± 5.48▲	123.04 ± 162.04▲
3 months	3.95 ± 0.73▲	84.27 ± 42.71▲	3.46 ± 4.09▲	72.14 ± 56.27▲
6 months	3.55 ± 1.33▲	63.84 ± 22.58▲	4.47 ± 3.12▲	65.88 ± 17.58▲

Supplementary Table 4: Blood pressures post-adrenalectomy

	Prednisone		Hydrocortisone	
	systolic (mm Hg)	diastolic (mm Hg)	systolic (mm Hg)	diastolic (mm Hg)
admission	145.90 ± 12.65	89.45 ± 14.38	149.27 ± 8.62	94.41 ± 7.20
discharge	131.55 ± 13.18▲	85.05 ± 9.68	134.64 ± 14.27▲	85.36 ± 9.78▲
1 month	127.65 ± 7.33▲	83.1 ± 5.08▲	129.36 ± 13.70▲	79.27 ± 7.73▲
3 months	122.60 ± 6.48▲	78.8 ± 5.48▲	123.05 ± 11.89▲	78.81 ± 7.40▲
6 months	119.10 ± 10.24▲	72.00 ± 6.62▲	116.82 ± 10.90▲	77.95 ± 7.75▲

Supplementary Table 5: Electrolytes post-adrenalectomy

Stage	Prednisone		Hydrocortisone	
	sodium mmol/L	potassium mmol/L	sodium mmol/L	potassium mmol/L
admission	144.25 ± 5.29	3.41 ± 0.61	143.14 ± 3.24	3.52 ± 0.51
discharge	142.05 ± 2.33▲	4.01 ± 0.40▲	141.70 ± 2.31▲	3.79 ± 0.33▲
1 month	141.05 ± 2.89▲	4.10 ± 0.25▲	141.43 ± 2.19▲	3.81 ± 0.35▲
3 months	140.80 ± 3.09▲	4.23 ± 0.34▲	141.33 ± 2.47▲	3.97 ± 0.38▲
6 months	140.09 ± 3.88▲	4.07 ± 0.24▲	140.88 ± 3.24▲	3.96 ± 0.27▲

Supplementary Table 6: Heart rate and blood glucose post-adrenalectomy

Stage	Prednisone		Hydrocortisone	
	Heart rate per minute	FPG mmol/L	Heart rate per minute	FPG mmol/L
admission	81.70 ± 7.01	6.88 ± 2.61	81.91 ± 12.01	6.57 ± 2.95
discharge	81.00 ± 8.42	5.93 ± 1.52	75.91 ± 9.05	5.73 ± 1.39
1 month	80.65 ± 5.40	5.29 ± 1.08▲	80.73 ± 7.04	4.83 ± 0.77▲
3 months	79.65 ± 6.32	4.96 ± 0.51▲	77.18 ± 3.63	4.55 ± 0.58▲
6 months	77.70 ± 5.07	4.96 ± 0.49▲	77.50 ± 5.74	4.38 ± 0.60▲

Note: raw data in the supplemental tables are presented in mean ± SD, then transformed to mean ± SEM for statistical analysis by normalizing to the sample size.
